# Intraregional differences in renal function in the Northern Netherlands: The Lifelines Cohort Study

**DOI:** 10.1371/journal.pone.0223908

**Published:** 2019-10-15

**Authors:** Qingqing Cai, Louise H. Dekker, Stephan J. L. Bakker, Martin H. de Borst, Gerjan Navis

**Affiliations:** 1 Department of Medicine, Division of Nephrology, University Medical Center Groningen, University of Groningen, Groningen, the Netherlands; 2 Aletta Jacobs School of Public Health, Groningen, The Netherlands; University of Glasgow, UNITED KINGDOM

## Abstract

**Background:**

Although the interregional disparity in chronic kidney disease (CKD) prevalence has been reported globally, little is known about differences in CKD prevalence within a region. We aimed to study the intraregional distribution of renal function in the Northern Netherlands and identify determinants of geographical differences in renal function.

**Methods:**

We included 143,735 participants from the Lifelines population-based cohort in the Northern Netherlands. Spatial analysis was performed to identify regional clusters of lower eGFR (cold spots) and higher eGFR (hot spots) at the postal code level, without and with adjustment for clinical risk factors. Multivariate logistic regression was used to identify the contribution of neighborhood-level health-related behaviors, socioeconomic status, and environmental factors (air pollution parameters, urbanity) to regional clustering of lower eGFR.

**Results:**

Significant spatial clustering of renal function was found for eGFR as well as for early stage renal function impairment (eGFR<90 ml/min/1.73 m^2^), (p<0.001). Spatial clustering persisted after adjustment of eGFR for clinical risk factors. In adjusted cold spots, the aggregate eGFR was lower (mean ± SD: 96.5±4.8 *vs*. 98.5±4.0 ml/min/1.73 m^2^, p = 0.001), and the prevalence of early stage renal function impairment (35.8±10.9 vs. 28.7±9.8%, p<0.001) and CKD stages 3–5 was higher (median (interquartile range): 1.2(0.1–2.4) vs 0(0–1.4)%, p<0.001) than in hot spots. In multivariable logistic regression, exposure to NO_2_ (Odd ratio [OR], 1.45; 95% confidence interval [95% CI], 1.19 to 1.75, p<0.001) was associated with cold spots (lower renal function), whereas proportion of fat intake in the diet (OR, 0.68; 95%CI, 0.48–0.97, P = 0.031) and income (OR, 0.91; 95%CI, 0.86–0.96, p<0.001) for median level income) were inversely related.

**Conclusions:**

Significant intraregional clustering of renal function, early renal function impairment and CKD were observed in the Northern Netherlands even after adjustment for renal function-related clinical risk factors. Environmental (air pollution), neighborhood-level socioeconomic factors and diet are determinants of intraregional renal function distribution. Spatial analysis might be a useful adjunct to guide public health strategies for the prevention of CKD.

## Introduction

Chronic kidney disease (CKD) is a global public health problem leading to considerable morbidity and mortality, with an increasing prevalence [1–2]. Patients with CKD are at increased risk of morbidity and premature mortality, mainly due to the increased prevalence of cardiovascular disease and infections. CKD may progress to end-stage kidney disease (ESKD) requiring dialysis or transplantation, and leads to substantial healthcare costs [3–5].

Regional differences in the prevalence of CKD have been reported globally. For example, in Europe, the reported prevalence of CKD stages 3–5 varies between 1.0% in central Italy and 5.9% in northeast Germany [6]. In the adult U.S. population, the prevalence of CKD stages 3–5 varies from 4.8% in the Northeast to 11.8% in the Midwest [7]. Similarly, in China the prevalence of CKD stages 3–5 varies from 1.1% in East China to 3.8% in Southwest China [8]. The observed differences in CKD prevalence across countries and regions can be explained by true discrepancies in CKD prevalence, but also due to heterogeneity of studies and methodology [6]. Analysis of factors driving regional variation might be useful to guide prevention strategies. Yet, the application of spatial analysis as a dissection tool has so far been limited, amongst others by the relatively coarse geographic scales of currently available data, whereas emerging evidence emphasizes the importance of regional variation at smaller spatial scales, such as neighborhood variance [9].

People living in the same community or neighborhood to some extent experience similar exposures and health status due to a common environment [10]. Emerging evidences show that living in socioeconomically disadvantaged neighborhoods is related to higher rates of disease, such as diabetes, cardiovascular disease and ESKD incidence [11–12]. Health interventions and policies may be less effective when neighborhoods factors are not taken into account [13]. Consequently, there is an increasing interest in evaluating health problems at the neighborhood level, and analyze differences on an intraregional scale. Identification of intraregional distribution of renal function could guide the medical community and policymakers to focus on allocating the limited resources effectively. Therefore, we investigated the spatial distribution of renal function at postal code level in the Lifelines cohort in the Northern Netherlands, a region comprising 9,315 km2 and 986 postal codes, with an even distribution of cohort participants over the region, and aimed to identify determinants of the geographic variation in renal function. To this purpose, we first performed spatial analysis on crude eGFR, thus identifying regional clusters of lower (cold spots) and higher (hot spots) eGFR. Next, to assess whether spatial distribution of eGFR might be explained by the spatial distribution of renal function-related risk factors, we performed spatial analysis for eGFR adjusted for known clinical risk factors. Finally, we identified determinants for the identified adjusted cold and hot spots by multivariate logistic regression, investigating the possible contribution of neighborhood-level health-related behaviors, socioeconomic status, and environmental factors, i.e air pollution parameters and urbanity.

## Methods

### Lifelines Cohort Study

Lifelines is a population-based cohort in a three-generation design to study the health and health-related behaviors of more than 165,000 participants living in the Northern Netherlands, accounting for 10% of the population, evenly distributed over the region. Participants were recruited from 2006 and 2011 through invitation by their general practitioners in the three Northern provinces of the Netherlands (Groningen, Friesland and Drenthe), and subsequently included their children, and parents, if available. In addition, inhabitants of the Northern provinces, who were not invited by their general practitioner, could register themselves via the Lifelines website. Detailed information about the Lifelines Cohort Study can be found elsewhere [14]. The Lifelines adult study population is broadly representative with respect to socioeconomic characteristics, lifestyle factors, the prevalence of chronic diseases and general health in the Northern Netherlands [15]. Before entering the study, all participants signed the informed consent. The Lifelines Cohort Study is conducted according to the principles of the Declaration of Helsinki. Our research protocol and data access application were reviewed and approved by the medical ethical review committee of the University Medical Center Groningen.

**Fig 1:**
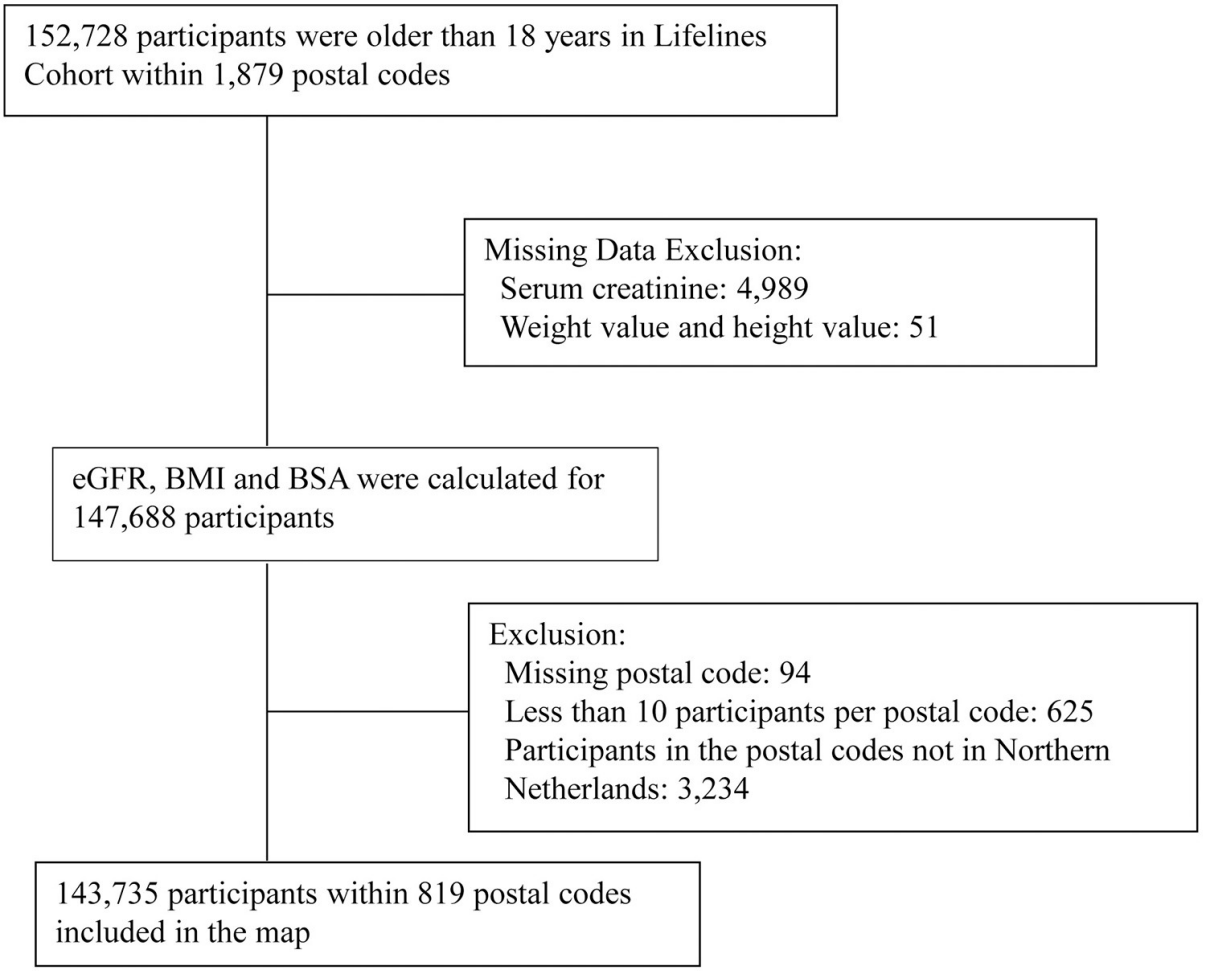
Flow chart of participants selection.

In the Lifelines cohort, 152,728 participants were older than 18 years. After excluding the missing clinical data on serum creatinine, weight and height, estimated glomerular filtration (eGFR), body mass index (BMI) and body surface area (BSA) data were calculated for 147,688 participants. Due to 94 participants with missing postal codes, 625 participants whose postal code area included less than 10 participants, and 3,234 participants who were not living in the Northern Netherlands, a total of 143,735 participants remained for the current analysis. Fig 1 shows flow diagrams outlining the study population, exclusions, and missing data.

### Sociodemographic characteristics and health-related behaviors

Sociodemographic characteristics and health-related behavior data were assessed based on self-administered questionnaires. Education level was classified into four groups (low: never been to school or elementary school only or lower vocational or secondary school; median: intermediate vocational school or intermediate/higher secondary school; high, higher vocational school or university; unknown or no answer. Income level was defined as a mean gross monthly income of: low: <1,000 euro; median: 1,000–3,000 euro; high: >3,000 euro; unknown or no answer. Smoker was defined as current smokers. Physical activity was evaluated by the validated Short Questionnaire to Assess Health-enhancing physical activity (SQUASH) questionnaire. In this study, we evaluated the moderate to vigorous physical activity. Self-administered food frequency questionnaire (FFQ) were used to assess proportions of the total protein intake, carbohydrate intake, fat intake in the diet, and total energy intake in this study [14,16].

### Clinical factors

Following a standardized protocol, trained technicians measured each individual’s height and weight, waist circumferences, and blood pressure. Body mass index (BMI) was calculated as weight (kg) divided by height squared (m^2^). Body surface area (BSA) was calculated by the Du Bois formula: *BSA* = 0.007184 × *Weight*^0.425^ × *Height*^0.725^. Biochemistry and renal function assessment included blood laboratory assessment and urine laboratory assessment. Estimated glomerular filtration rate (eGFR) was calculated using the Chronic Kidney Disease Epidemiology Collaboration equation (CKD-EPI) [17]. Early stage renal function impairment was defined as eGFR < 90 ml/min/1.73m^2^. CKD stages 3–5 was defined as eGFR <60 ml/min/1.73m^2^. Participants were categorized as having diabetes if they had self-reported diabetes and/or a non-fasting plasma glucose ≥11 mmol/L and/or a measured glycated hemoglobin (HbA1c) ≥6.5% (48 mmol/mol) and/or use of oral anti-diabetics and/or insulin. Cardiovascular disease included coronary artery disease, heart failure and/or stroke. Hypertension was defined as blood pressure >140/90 mmHg or use of anti-hypertensive medication.

### Environmental factors

Urbanity and air pollution were included as environmental factors in this study. The degree of urbanity was obtained from Statistics Netherlands (CBS) 2011 data [18]. There were three categories of urbanity: rural, <500 addresses per km^2^; semi-urban, 500–1,500 addresses per km^2^; urban, >1,500 addresses per km^2^. Two air pollutants were included in the present study. Annual average concentration of NO_2_ and fine particulate matter with aerodynamic diameter <2.5 μm (PM_2.5_) were estimated at place of residence for the periods 2009–2010 using the European Study of Cohort for Air Pollution Effects (ESCAPE) land-use regression (LUR) model incorporating satellite-derived and chemical transport modelling data, which have been described elsewhere [19–21].

### Neighborhoods and neighborhood-level factors

Neighborhood was defined by postal code in this study. The Northern Netherlands comprises a total of 986 postal codes. Participants resided in 1,879 postal codes in Lifelines cohort. After excluding postal codes not in the Northern Netherlands and postal codes with less than 10 participants, a total of 819 postal codes were included in our study (Fig 1). The mean number of participants per postal code in our study was 176 (median 69). The value of neighborhood-level factors was aggregated as the median value of individual factors at each postal code.

### Spatial analysis

Spatial analysis was applied to identify clusters of neighborhoods with higher or lower renal function and early stage of renal function impairment. Global Moran’s I (GMI) statistic is a commonly used measure of spatial autocorrelation based on a predefined spatial neighborhood [22]. The index of the GMI ranges between -1 and 1, indicating from maximum negative association to maximum positive association. A significantly positive GMI means spatial clustering takes place. A significantly negative GMI is called dispersion. A zero value indicates a random spatial distribution. A higher positive value means a stronger spatial autocorrelation, and vice versa for negative values. The level of significance is set at p<0.05. We applied this method to identify unadjusted eGFR and adjusted eGFR clustering at postal code level in ArcGIS v10.3.

Global measures of spatial association identify whether clustering takes place across the study area. However, GMI does not show where the clusters occur. Therefore, Getis-Ord Gi* Hot Spot Analysis was used in this study to detect spatial clusters. This method was used in Lifelines cohort previously [23]. The rationale behind the Gi* is that for each area i, a weighted average is constructed for the variables, using the value for area i with a weight of 1, and the values of areas surrounding i weighted by the inverse distance to i. The resultant weighted average is normalized and can then be interpreted as a z-score. Positive z indicated “hot spots” (red) where postal codes with higher values are near neighboring postal codes with higher values, negative z indicated “cold spots” (blue) where postal codes with lower values are near neighboring postal codes with lower values. And then the clusters mapped according to the p-value related to z-score (p<0.01 mapped in dark blue or red, p<0.05 mapped in medium blue or red, and p<0.1 mapped in light blue or red). We identified statistically significant hot spots and cold spots at the postal code level using the Getis-Ord Gi* statistic in ArcGIS v10.3.

### Statistical analysis

The aggregate eGFR value per postal code was used to perform GMI and Getid-Ord GI*. The adjusted eGFR per postal code was first obtained from individual eGFR adjusted for known renal function-related clinical risk factors by linear regression, and then the derived residuals were aggregated by postal codes. The adjusted clinical risk factors included age, sex, BSA, BMI, waist circumference, serum potassium, cholesterol, triglycerides, diabetes, cardiovascular disease, hypertension. In order to assess the determinants of the clustering of renal function, we compared neighborhood-level health-related behaviors, socioeconomic status, and environmental factors between adjusted cold and hot spots at 90, 95 and 99% confidence levels. Univariable and multivariable logistic regression was applied to identify the neighborhood-level factors associated with being located in cold spots compared to hot spots. Statistical analyses were performed by SPSS (version 25.0, Armonk, NY: IBM Corp).

## Results

Individual-level subject characteristics of the overall cohort (n = 143,735) and adjusted cold and hot spots are given in S1 Table. Mean (mean ± standard deviation) age was 44.8±13.0 years. The age range was 18–93 years. Fifty-eight percent of the participants were women. The prevalence of early stage renal function impairment (eGFR<90 ml/min/1.73m^2^) was 32.4%, and the overall prevalence of CKD stages 3–5 was 1.2%.

### Intraregional renal function distribution

**Fig 2:**
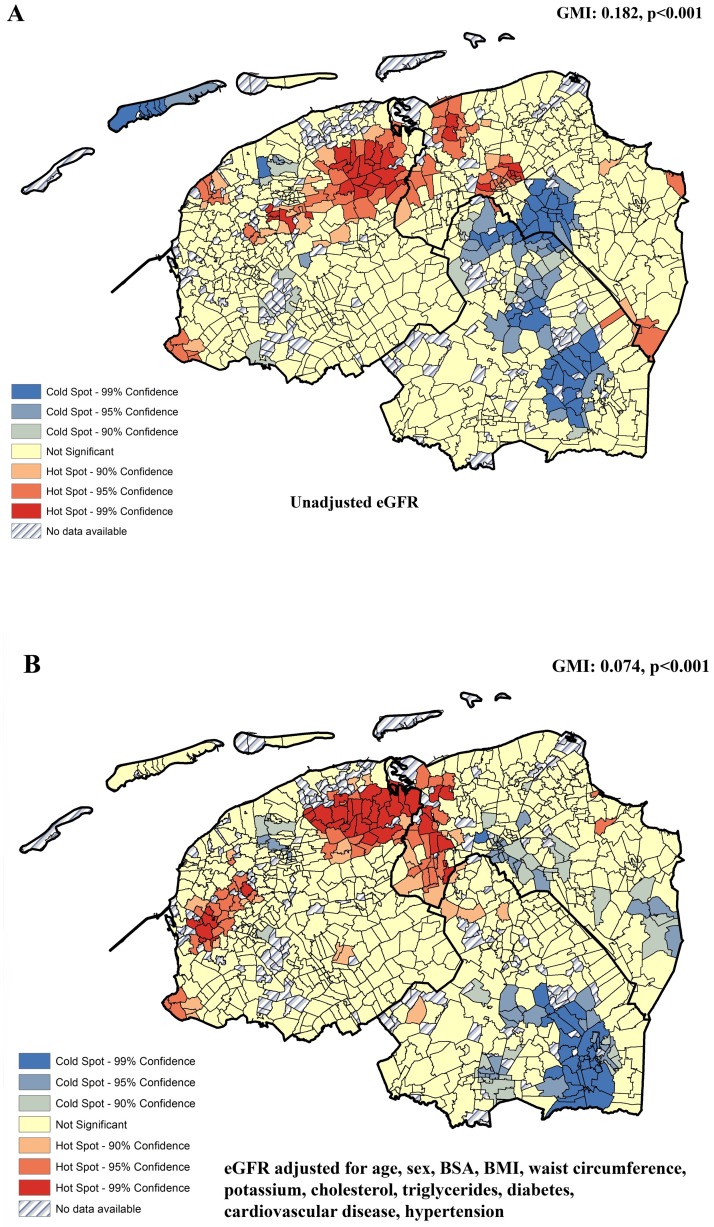
The intraregional distribution of eGFR-based renal function in Northern Netherlands. (A) Hot (red colors) and cold spot (blue colors) clusters of unadjusted eGFR. (B) Hot (red colors) and cold spot (blue colors) clusters of eGFR adjusted for known clinical risk factors, including age, sex, BSA, BMI, waist circumference, serum potassium, cholesterol, triglycerides, diabetes, cardiovascular disease and hypertension.

**Fig 3:**
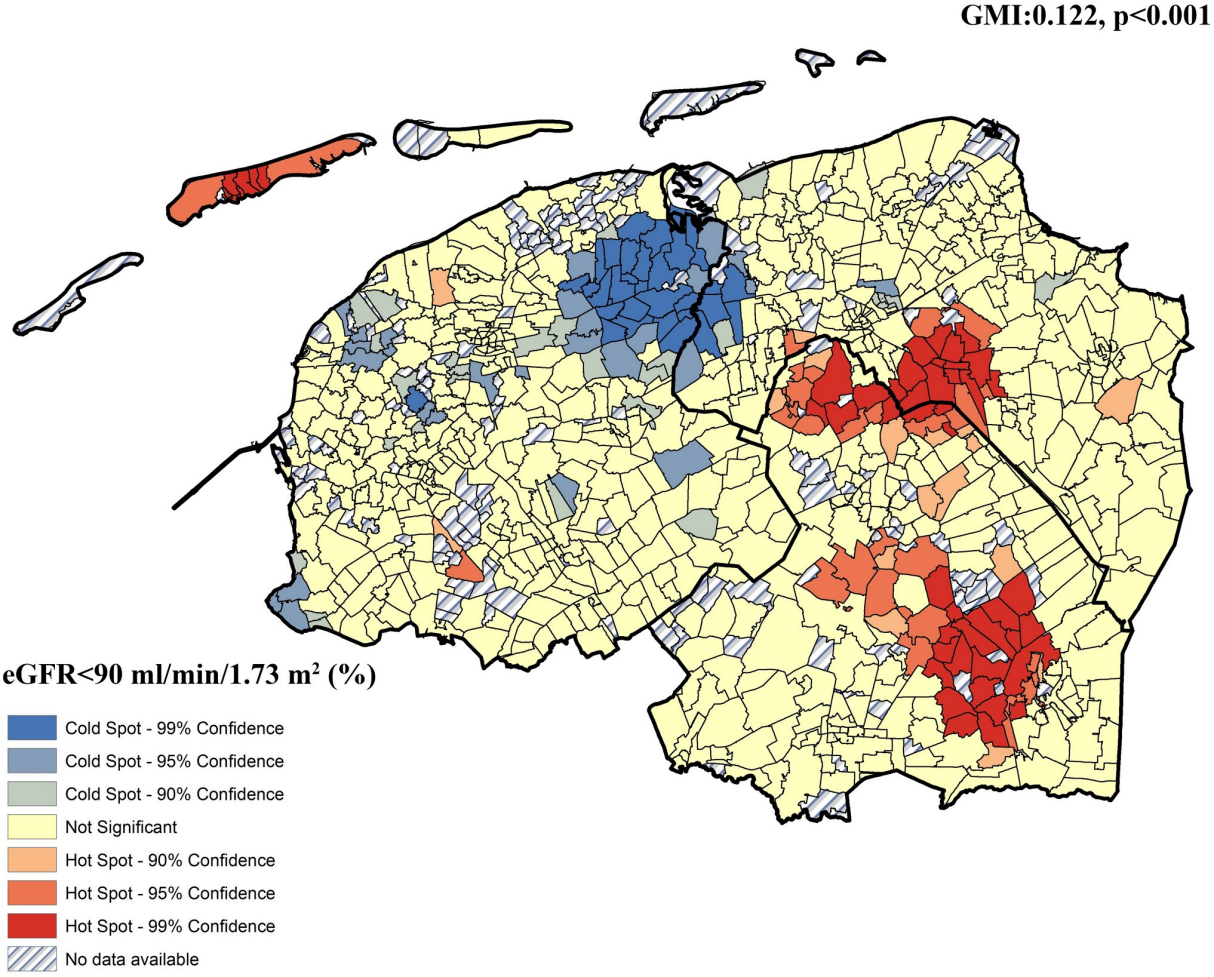
The intraregional distribution of proportion of eGFR<90 ml/min/1.73m^2^ in Northern Netherlands. Hot spots were shown as red colors and cold spot were shown as blue colors.

Significant regional clustering was found, for both unadjusted (GMI:0.182, p<0.001) and adjusted eGFR (GMI:0.074, p<0.001), as illustrated in Fig 2A and 2B, showing clusters of neighborhoods with higher eGFR (hot spots) and with lower eGFR (cold spots). Adjustment for known renal function-related clinical risk factors (Fig 2B), significant clusters of cold and hot spot persisted (GMI:0.074, p<0.001), indicating that the regional clustering of renal function cannot be fully explained by established clinical risk factors of renal function. For early stage renal function impairment (eGFR<90 ml/min/1.73 m^2^ (%), significant regional clustering was detected as well, as illustrated in Fig 3 (GMI:0.122, p<0.001).

### Neighborhood-level characteristics of adjusted cold and hot spots

Neighborhood-level characteristics of the overall cohort (819 postal code areas) as well as the adjusted cold (109 postal code areas) and hot (114 postal code areas) spots are shown in Table 1. In the adjusted cold spots, the aggregate eGFR (96.5±4.8 vs. 98.5±4.0 ml/min/1.73 m^2^, p = 0.001) and 24-hour creatinine clearance (122.8±5.9 vs. 127.6±8.6 ml/min, p<0.001) were lower than in hot spots. In line, the prevalence early stage renal function impairment was higher (35.8±10.9 vs. 28.7±9.8%, p<0.001) as was the prevalence of CKD stages 3–5 (median (interquartile range): 1.2(0.1–2.4) vs 0(0–1.4) %, p<0.001). Age was lower, serum potassium was lower, and BMI and waist circumference were higher in the adjusted cold spots. Other clinical risk factors were not different. In adjusted cold spots, proportion of fat consumption was lower than in adjusted hot spots (p<0.001). Socioeconomic status showed differences for education level, with an overrepresentation of higher education in the adjusted cold spots, as well as income level, with overrepresentation of low income and high income, and underrepresentation of median level income in the adjusted cold spots, as compared to adjusted hot spots. As to urbanity, 33.9% of the adjusted cold spots were in urban regions, as compared to 4.4% for the hot spots. (p = 0.001). Exposure to NO_2_ and PM_2.5_ was higher in adjusted cold spots than in adjusted hot spots (p<0.001).

**Table 1:** Cohort characteristics at neighborhood level (overall and for adjusted cold and hot spots).

	Overall cohort (aggregate value) ^1^	Cold spots (aggregate value)	Hot spots (aggregate value)	P values^2^
Number	819	109	114	
**Demographics**				
Age (year)	45.7±4.1	44.1±6.1	45.9±3.8	**0.010**
Sex, female (%)	58.3±5.4	58.8±4.3	58.7±5.9	0.877
**Clinical factors**				
Body surface area (m^2^)	1.9±0.1	1.9±0.1	1.9±0.1	0.583
24h urine creatinine clearance (ml/min)	124.9±7.0	122.8±5.9	127.6±8.6	**<0.001**
Serum creatinine (μmol/L)	72.0±2.4	72.9±2.4	70.7±2.7	**<0.001**
eGFR (ml/min/1.73 m^2^)	97.0±4.0	96.5±4.8	98.5±4.0	**0.001**
eGFR<90 ml/min/1.73 m^2^ (%)	32.9±9.6	35.8±10.9	28.7±9.8	**<0.001**
CKD stages 3–5 (%)	0.8(0–1.7)	1.2(0.1–2.4)	0(0–1.4)	**<0.001**
Serum potassium (mmol/L)	3.9±0.1	3.8±0.1	3.9±0.1	**<0.001**
BMI (kg/m^2^)	25.5±0.9	25.6±1.2	25.2±0.8	**0.023**
Waist circumference (cm)	89.5±3.2	89.6±3.9	88.9±2.7	**0.088**
Cholesterol (mmol/L)	5.1±0.2	5.0±0.2	5.1±0.2	0.101
Triglycerides (mmol/L)	1.0±0.1	1.0±0.1	1.0±0.1	0.103
Diabetes (%)	3.0(1.0–4.7)	3.2(1.8–5.1)	2.7(0–4.4)	0.079
Hypertension (%)	21.1±7.1	20.4±7.2	21.0±7.5	0.547
Cardiovascular disease (%)	2.6(0.9–4.2)	2.9(1.6–4.1)	2.5(0–4.3)	0.161
**Health-related behaviors**				
Smoker (%)	18.2±6.9	18.3±7.1	18.8±7.6	0.647
Physical activity (min/week)	360(265–440)	435(378–540)	390(279–585)	0.144
Total protein intake (g/day/1000kcal)	38.2±5.7	37.3±4.0	36.8±3.2	0.325
Total carbohydrate (g/day/1000kcal)	109.8±6.8	111.1±5.4	111.2±4.6	0.896
Total fat intake (g/day/1000kcal)	39.2±2.0	39.1±1.2	39.8±1.3	**<0.001**
Total energy intake (kcal)	1881±466	1901±252	2027±289	**<0.001**
**Socioeconomic status Education (%)**				
Low	29.6±10.7	28.3±13.0	32.9±10.5	**0.005**
Median	39.7±9.5	38.6±9.9	42.6±11.0	**0.005**
High	28.3±12.6	32.5±15.9	24.0±10.7	**<0.001**
Unknown/no answer	2.3±2.2	0.5±0.7	0.5±1.2	0.395
**Income (%)**				
Low	5.7±4.5	8.4±6.9	5.2±3.7	**<0.001**
Median	46.0±11.6	45.4±10.5	50.5±10.5	**<0.001**
High	28.2±12.4	27.6±11.5	24.5±10.1	**0.037**
Unknown/no answer	20.0±9.1	18.7±8.1	19.8±8.6	0.296
**Environmental factors Urbanity (%)**^**3**^				
Rural	65.8	46.8	84.1	**0.001**
Semi-urban	15.3	19.3	11.5	
Urban	18.9	33.9	4.4	
**Air pollution (ug/m**^3^**)**				
NO_2_	18.3±4.0	21.4±5.4	16.8±2.4	**<0.001**
PM_2.5_	14.4±0.9	14.8±0.8	14.1±0.8	**<0.001**

^1^ Neighborhood characteristics are presented as aggregate value, that were calculated as follows: medians were calculated per postal code area, and the average (±SD) of the medians is presented in the table.

^2^ P values: comparison between cold and hot spots; p<0.05 presents statistical significance

^3^ Urbanity is a neighborhood-level variable, others are the aggregate value of individuals in each postal code.

Definitions: Education: low: never been to school or elementary school only or lower vocational or secondary school; median: intermediate vocational school or intermediate/higher secondary school; high, higher vocational school or university. Income: low, < 1,000euro; median, 1,000–3,000 euro; high, >3,000 euro. Urbanity: rural, <500 addresses per km^2^, semi-urban, 500–1,500 addresses per km^2^; urban, >1,500 addresses per km^2^.

### Determinants of renal function distribution

Univariate and multivariate logistic regression analysis were conducted on adjusted cold and hot spots to identify their neighborhood-level determinants. The results are given in Table 2. In univariate logistic regression, high education, low and high income, urbanity and air pollution was positively associated with being in cold spots (all p<0.05), whereas proportion of total fat intake in the diet, low and median education, and median income showed inverse associations with being in cold spots (all p<0.05). In final multivariate logistic regression model (model 3), NO_2_ (Odds ratio [OR], 1.45; 95% confidence interval [95% CI], 1.19 to 1.75, p<0.001) was positively associated with being in the adjusted cold spots, while proportion of total fat intake in the diet (OR,0.68; 95%CI, 0.48–0.97, p = 0.031) and median level income (OR,0.91; 95%CI, 0.86–0.96, p<0.001) were negatively associated. Notably, the association with education lost significance after adjustment. The association with urbanity lost significance when adjusted for air pollution. Taken together, neighborhood-level socioeconomic status, i.e income level, and diet, i.e proportion of fat intake, and environmental factors, i.e NO_2_ exposure were associated with spatial clustering of eGFR, with lower proportion of fat intake, median level income and higher NO_2_ exposure being related to adjusted cold spots (lower eGFR).

**Table 2:** Association between neighborhood-level health-related behaviors, socioeconomic status and environmental factors and adjusted cold spots by logistic regression.

	Univariate logistic regression			Multilvariate logistic regression								
				Model 1			Model 2			Model 3		
	OR	95%CI	P	OR	95%CI	P	OR	95%CI	P	OR	95%CI	P
**Health-related behaviors**												
Smoker (%)	0.99	0.96–1.03	0.645	0.99	0.95–1.04	0.856	0.98	0.93–1.03	0.452	0.97	0.91–1.02	0.260
Physical activity (min/week)	1.01	0.99–1.01	0.546	1.01	0.99–1.01	0.192	1.01	0.99–1.01	0.231	1.01	0.99–1.01	0.238
Total protein intake (g/day/1000kcal)	1.04	0.95–1.14	0.354	0.98	0.79–1.20	0.821	0.95	0.77–1.17	0.635	0.93	0.74–1.17	0.548
Total carbohydrate (g/day/1000kcal)	0.99	0.95–1.05	0.895	1.02	0.87–1.15	0.966	0.96	0.83–1.11	0.583	0.94	0.80–1.10	0.439
Total fat intake(g/day/1000kcal)	0.59	0.43–0.78	**<0.001**	0.65	0.47–0.89	**0.008**	0.68	0.49–0.94	**0.019**	0.68	0.48–0.97	**0.031**
**Socioeconomic status Education (%)**												
Low	0.97	0.95–0.99	**0.006**	0.94	0.69–1.30	0.725	1.01	0.72–1.39	0.987	1.04	0.74–1.45	0.835
Median	0.96	0.94–0.99	**0.006**	0.91	0.66–1.25	0.568	0.98	0.71–1.35	0.879	1.01	0.71–1.40	0.997
High	1.04	1.03–1.07	**<0.001**	0.94	0.68–1.30	0.717	0.99	0.71–1.38	0.966	1.03	0.73–1.45	0.870
**Income (%)**												
Low	1.13	1.06–1.20	**<0.001**	1.15	1.05–1.25	**0.002**	1.08	0.98–1.19	0.105	1.02	0.92–1.15	0.671
Median	0.95	0.93–0.98	**0.001**	0.95	0.91–0.99	**0.030**	0.3	0.89–0.98	**0.004**	0.91	0.86–0.96	**<0.001**
High	1.03	1.01–1.05	**0.038**	1.01	0.96–1.06	0.636	0.99	0.94–1.04	0.769	0.96	0.91–1.02	0.173
**Environmental factors Urbanity (%)**												
Rural	reference			-	-	-	reference			reference		
Semi-urban	3.01	1.39–6.05	**0.005**	-	-	**-**	3.03	1.31–7.01	**0.009**	0.69	0.24–1.97	0.492
Urban	13.78	5.10–37.24	**<0.001**	-	-	**-**	9.41	2.95–29.99	**<0.001**	0.48	0.09–2.52	0.385
**Air pollution (ug/m**^**3**^**)**												
NO_2_	1.36	1.23–1.50	**<0.001**	-	-	**-**	-	-	**-**	1.45	1.19–1.75	**<0.001**
PM_2.5_	2.86	1.95–4.20	**<0.001**	-	-	**-**	-	-	**-**	1.38	0.79–2.43	0.256

Model 1: adjusted for health-related behaviors and socioeconomic status.

Model 2: model1 plus urbanity

Model 3: model 2 plus air pollution

## Discussion

In this study, we found significant spatial differences in renal function and the prevalence of early stage renal function impairment as well as the prevalence of CKD stages 3–5. The spatial distribution was not explained by the known renal function-related clinical risk factors. The neighborhood-level factors, NO_2_, income and lower proportion of fat intake, were independently related to clusters of lower renal function after adjustment. This is the first study to identify intraregional renal function difference at the neighborhood level in The Netherlands and to identify explanatory factors, namely diet, income level and air pollution. These findings provide a basis for better-targeted preventive and public health measures in the prevention of renal function decline and CKD.

The spatial analysis in this report suggested the presence of regions in which the renal function is higher and lower, respectively, than could be expected if the renal function would be randomly distributed over the Northern Netherlands. A similar variation within regions was recently reported within the United States and France. Here, Bowe at al. examined the geographic variation of kidney function decline among U.S. counties, and identified county characteristics associated with rapid kidney function decline. However, the authors did not identify factors responsible for these clusters [24]. And Occelli at al. mapped the end-stage kidney disease (ESKD) on small area level in Northern France and revealed significant geographic difference in ESKD incidence [25]. In agreement with US and France study, we found relatively consistent renal function distribution with or without adjustment of renal function-related clinical risk factors. The results showed that regions and geographic factors still matter among individuals within the homogeneous (national) health care system, indicating the importance of taking these factors into account in allocating health care resources. The GMI statistic as a geographic tool can be a useful adjunct to guide strategies for prevention of renal function decline and CKD at a local scale. Even when known clinical risk factors are taken into account, spatial disparities in renal function remain, suggesting that neighborhood-level factors may play important roles.

Accordingly, we identified several neighborhood-level determinants of cold and hot spots of adjusted eGFR. As to neighborhood-level health behaviors, lower fat intake was associated with adjusted cold spots on multivariate analysis. The association between fat intake and renal function is complex, and may relate to type of fat, presence of pre-existing renal and/or albuminuria [26], and concomitant cardiovascular and metabolic derangements. Our data may seem at variance with the literature, as a higher proportion of fat in the diet was associated with a lower risk of being in a cold spot. However, it should be noted that we analyzed neighborhood-level diet intake, as opposed to the individual-level analysis in the literature which precludes a direct comparison. Our study design, and lack of data on type of fat, does not allow to inferences as to possible mechanisms, yet, it is relevant to note that the association was present for adjusted eGFR clustering, thus pointing towards effects of neighborhood-level fat intake independent from the known clinical risk factors. The association of diet-related factors like fat intake suggests that regional differences in dietary habits, as previously reported in the Lifelines cohort [23], may be relevant to geographic clustering of renal function, but this assumption would require further study to substantiate.

We found that, neighborhood-level income was independently associated with the risk of being in the cold spots, with the lowest risk for median level income, whereas the lower risk in the higher income group (OR 0.96; 95% CI 0.91–1.02) feel short of statistical significance. Low socioeconomic status has previously been recognized as a risk factor for the incidence and progression of CKD [27], and both lower income and lower education were proven to be associated with renal function decline [28], mostly in individual-level studies. One report, from the US, compared the role of community-level poverty with individual-level poverty as a risk factor for end stage kidney disease and found that individual poverty was as risk factor, whereas community-level poverty was not [29]. This might be interpreted as being in contrast with our finding on neighborhood-level income as a determinant of clustering of lower renal function. However, our study was conducted in a very different setting, with also a different outcome parameter, precluding a direct comparison between the studies. Neighborhood-level education level was not associated with the clustering of renal function after adjustment for multiple risk factors. However, a previous study reported that education was associated with a less marked risk gradient than the occupational based socioeconomic status [30]. It seems income level is better to represent socioeconomic status than the education level.

Considering the spatial distribution of eGFR, the possible role of environmental factors is of specific interest. We found that urbanity was a risk factor, but this association lost significance after including air pollution in the model. Non-urbanity, and the consequent distance to specialist care, was a key factor affecting the outcomes of dialysis-dependent or non-dialysis dependent CKD patients in prior studies [31–32]. Remote dwelling patients were less likely to receive nephrologist care and had a higher risk of mortality or hospitalization [31]. We assume that distance to health care provisions is less of a problem in the Netherlands, where distances are small, and the health care system is accessible for all. The role of air pollution. i.e exposure to NO_2_ as an independent risk factor for being in a cold spot is of interest. Air pollution has previously been reported as a potential explanation of the geographical variation of kidney disease [33]. Previous studies found that chronic exposure to air pollution, including PM_2.5_, PM_10_, NO_2_ and CO, is a significant risk factor for the incident CKD and progression to ESKD [34–35]. Our study found that NO_2_ exposure was worse in the cold spots, i.e where renal function was worse, suggesting that air pollution should be considered an actionable risk factor for prevention of renal damage.

What could be the clinical significance of our findings? The differences in renal function between the adjusted hot and cold spots were relatively mild, but were accompanied by a corresponding difference in early renal function impairment, as well as CKD stages 3–5, which enhances the robustness of our findings. Within the entire cohort, the overall prevalence of CKD stage 3–5 was 1.2%. The aggregate prevalence of CKD in hot spots was 0 (interquartile: 0–1.4) % and in cold spots was 1.2 (0.1–2.4) %. The prevalence of CKD stages 3–5 has been reported to vary all over the world. Even within European countries it varies between countries, with prevalence ranging between 1.0% and 5.9% [6]. Although measurements of creatinine have been standardized, some interlaboratory variability still exists [36]. In the Lifelines cohort, for all samples, the same methodology was used to measure creatinine, so renal function distribution reflected true differences in CKD prevalence in cold and hot spots. The low prevalence of CKD may be explained by the good health care access in the Netherlands. Follow-up studies will have to show whether being in a cold spot also predisposes to progressive renal function loss. Yet, an important message is that clinical risk factors alone cannot explain the spatial distribution of renal function, and that, accordingly, better prevention of CKD requires public health measures. Our findings suggest that air pollution, and poverty, are relevant targets.

A strength of this study is that it is the first to use spatial analysis to visualize and detect the patterns of renal function and identify the neighborhood-level factors associated with renal function distribution in a large representative sample in the Netherlands. This study also has several limitations. First, due to its cross-sectional nature, this study could not account for changes in the distribution of renal function and its determinants over time. Second, although the analyses were adjusted for known renal function-related clinical risk factors, it is possible that unmeasured or unknown determinants and confounders could not be adjusted for in our study. Third, the Northern Netherlands is a low prevalence area for renal function impairment [6], this might limit the generalizability of our findings. Finally, it should be noted that for analysis of the contribution of neighborhood-level factors on risk of being in a cold spot, we used the aggregated values. This limits their interpretation, could elicit ecological fallacy, and in particular limits direct comparison with studies on the role of individual risk factors on renal function risk.

Significant variation in CKD prevalence are observed between regions and countries globally. In the present study, clustering of eGFR-based renal function was observed within a single region in the Northern Netherlands. The neighborhood-level diet, income and NO_2_ were identified as region-specific determinants for renal function clusters. More attention should be paid on neighborhood-level determinants like health-related behaviors, socioeconomic status and environmental factors. These data could advance our understanding of the neighborhood determinants of health and guide better prevention strategies.

## Supporting information

S1 Table Cohort characteristics at individual level (overall and for adjusted cold and hot spots). (DOCX)
