# Supplementary material for: Intraregional differences in renal function in the Northern Netherlands: The Lifelines Cohort Study
Source: PLoS One. 2019 Oct 15;14(10):e0223908. doi: 10.1371/journal.pone.0223908 (PMC6793867; doi:10.1371/journal.pone.0223908)
Supplement: S1 Table — (DOCX) [file pone.0223908.s001.docx]

# Supporting information

**S1 Table. Cohort characteristics at individual level (overall and for adjusted cold and hot spots)**

|  | **Overall cohort** | **Cold spots** | **Hot spots** | **P values^1^** |
| --- | --- | --- | --- | --- |
| Number | 143,735 | 26,372 | 13,567 |  |
| **Demographics** |  |  |  |  |
| Age (year) | 44.8±13.0 | 43.3±14.3 | 45.6±12.6 | **<0.001** |
| Sex, female (%) | 58.3 | 58.6 | 57.5 | **0.027** |
| **Clinical factors** |  |  |  |  |
| Body surface area (m^2^) | 1.9±0.2 | 1.9±0.2 | 1.9±0.2 | **<0.001** |
| 24h urine creatinine clearance (ml/min) | 126.9±35.0 | 125.6±35.4 | 128.7±33.6 | **<0.001** |
| Serum creatinine (µmol/L) | 73.5±13.7 | 73.9±13.8 | 72.7±13.7 | **<0.001** |
| eGFR (ml/min/1.73 m^2^) | 96.6±15.3 | 97.1±16.0 | 97.0±15.0 | 0.522 |
| eGFR<90 ml/min/1.73 m^2^ (%) | 32.4 | 31.9 | 30.8 | **0.034** |
| CKD stages 3-5 (%) | 1.2 | 1.5 | 1.1 | **0.002** |
| Serum potassium | 3.9±0.3 | 3.8±0.3 | 3.9±0.3 | **<0.001** |
| BMI (kg/m^2^) | 26.1±4.3 | 25.8±4.4 | 25.9±4.1 | 0.082 |
| Waist circumference (cm) | 90.3±12.5 | 89.7±12.8 | 89.8±12.0 | 0.227 |
| Cholesterol (mmol/L) | 5.1±1.0 | 5.0±1.0 | 5.1±1.0 | **<0.001** |
| Triglycerides (mmol/L) | 1.2±0.8 | 1.2±0.8 | 1.2±0.8 | 0.135 |
| Diabetes (%) | 3.5 | 3.5 | 3.4 | 0.471 |
| Hypertension (%) | 21.1 | 19.0 | 22.2 | **<0.001** |
| Cardiovascular disease (%) | 3.0 | 3.0 | 3.0 | 0.967 |
| **Health-related behaviors** |  |  |  |  |
| Smoker (%) | 18.9 | 19.8 | 17.9 | **<0.001** |
| Physical activity (min/week) | 360(130-900) | 425(180-740) | 390(120-753) | **<0.001** |
| Total protein intake (g/day/1000kcal) | 41.3±16.6 | 38.0±9.4 | 37.4±7.4 | **<0.001** |
| Total carbohydrate (g/day/1000kcal) | 108.4±19.6 | 111.7±15.8 | 111.9±14.3 | 0.224 |
| Total fat intake (g/day/1000kcal) | 38.3±7.6 | 38.7±6.0 | 39.4±5.6 | **<0.001** |
| Total energy intake (kcal) | 1881±847 | 1989±697 | 2088±681 | <0.001 |
| **Socioeconomic status** |  |  |  |  |
| **Education (%)** |  |  |  |  |
| Low | 30.1 | 27.0 | 31.7 | **<0.001** |
| Median | 39.9 | 37.2 | 41.3 |  |
| High | 29.5 | 35.1 | 26.5 |  |
| Unknown/no answer | 0.6 | 0.7 | 0.5 |  |
| **Income (%)** |  |  |  |  |
| Low | 6.7 | 10.3 | 5.6 | **<0.001** |
| Median | 47.3 | 47.0 | 48.7 |  |
| High | 27.7 | 27.7 | 28.0 |  |
| Unknown/no answer | 18.3 | 18.3 | 17.8 |  |
| **Environmental factors** |  |  |  |  |
| **Urbanity (%)** |  |  |  |  |
| Rural | 29.7 | 14.2 | 44.6 | **<0.001** |
| Semi-urban | 28.6 | 31.9 | 29.3 |  |
| Urban | 41.8 | 53.9 | 26.0 |  |
| **Air pollution (ug/m^3^)** |  |  |  |  |
| NO_2_ | 21.4±4.8 | 24.6±5.4 | 19.1±3.3 | **<0.001** |
| PM_2.5_ | 14.8±1.0 | 15.2±0.8 | 14.5±0.8 | **<0.001** |

^1^ P values: comparison between cold and hot spots; p<0.05 presents statistical significance

Definitions: Education: low: never been to school or elementary school only or lower vocational or secondary school; median: intermediate vocational school or intermediate/higher secondary school; high, higher vocational school or university. Income: low, < 1,000euro; median, 1,000-3,000 euro; high, >3,000 euro. Urbanity: rural, <500 addresses per km^2^, semi-urban, 500-1,500 addresses per km^2^; urban, >1,500 addresses per km^2^.
